# Real World of Percutaneous Coronary Interventions in the Public Health System in Rio de Janeiro: How Can It Be Improved?

**DOI:** 10.5935/abc.20180227

**Published:** 2018-11

**Authors:** Stefano Garzon, Expedito E. Ribeiro

**Affiliations:** Instituto do Coração (INCOR), São Paulo, SP - Brazil

**Keywords:** Coronary Artery Disease, Percutaneous Coronary Intervention/economics, Mortality, Morbidity, Unified Health System/economics, Epidemiology

Cardiovascular diseases (CVD) are currently the leading cause of death in Brazil^1^ and in the world,^2^ with 80% of the cases^3^ occurring in low- and middle-income countries. It impacts these countries economies negatively,^4^ with reductions in the Gross Domestic Product (GDP), and increases in the burden on already precarious health care systems. The risk factors associated with CVD are largely preventable, and raising awareness^5^ and increasing access to primary health care for prevention^6^ are key factors for reducing events.

The present study examined mortality rates in patients who underwent percutaneous coronary interventions (PCI) for both stable coronary disease (SCD) and acute coronary syndromes (ACS) in the State of Rio de Janeiro Public Health System (SUS) from 1999 to 2010. It provides us with interesting data regarding mortality outcomes in such patients, dividing them by gender, age groups, and type of intervention (balloon coronary angioplasty, stenting with bare metal stents and primary PCI for STEMI). It has obvious limitations: it is a retrospective populational cohort; its data were extracted from different databases, and the information had to be paired (hospital admissions versus death certificates, which are not in the same dataset); the mortality outcome was death by any cause, and although the authors cite that the cause of death was divided into two groups (cardiovascular death and any other cause), it is not clear which data was used; there is no information regarding comorbidities, single vessel versus multivessel disease, or medications prescribed; and patients with more than one PCI were excluded.^7^

The authors also state that, compared with other studies,^8^^-^^10^ the present study showed higher mortality rates, attributing that to the difficulties of extrapolating randomized clinical trials (RCT) results to real-world practice. Although external validity of RCTs and generalizability of their results is a known issue,^11^ it is also reasonable to consider the precariousness of the Brazilian Public Health Care System (SUS), with restricted access to primary care and preventive medicine, unsteady supply of medication, unavailability of drug-eluting stents, and insufficient secondary and tertiary health care structure. Above all, low socio-economic conditions and education contribute to a scenario where there are many confounding factors to this higher mortality rates. We also have to consider that there is no evidence that PCI for SCD reduces mortality when compared to optimized medical treatment;^8^ therefore, perhaps a better primary outcome could be major cardiac and cerebrovascular events (MACCE) rather than death alone, although it is understandable that the lack of a unified registry, with thorough information, makes it virtually impossible.

Finally, it would be interesting to investigate the costs of cardiovascular disease to SUS, and to compare the financial burden of CVD in Brazil to that in other countries.^12^

Besides its limitations, the present study has strong points: a large number of individuals, a long follow-up time, and a real-world setting. It should be used to generate questions rather than providing answers, and it is a big step towards providing better care for our patients in Brazil.
